# Short-course vunakizumab associated with rapid improvement of acute flares in paediatric generalised pustular psoriasis: a three-patient case series

**DOI:** 10.3389/fimmu.2026.1890409

**Published:** 2026-09-18

**Authors:** Juan Huang, Yue Li, Yiqian Peng, Ping Xia, Feng Hu, Jinbo Chen

**Affiliations:** 1 Hubei University of Chinese Medicine, Wuhan, China; 2 Department of Dermatology, Traditional Chinese and Western Medicine Hospital of Wuhan, Hubei University of Chinese Medicine, Wuhan, China; 3 Department of Dermatology, Wuhan No.1 Hospital, Wuhan, China; 4 Tongji Medical College, Huazhong University of Science and Technology, Wuhan, China; 5 Hubei Province Key Laboratory of Skin Infection and Immunity, Wuhan, China; 6 Department of Dermatology, Hubei Provincial Hospital of Traditional Chinese Medicine, Hubei Province Academy of Traditional Chinese Medicine, Wuhan, China

**Keywords:** acute flare, case series, generalized pustular psoriasis, GPPASI, IL-17A, paediatric, short-course treatment, vunakizumab

## Abstract

**Background:**

Paediatric generalised pustular psoriasis (GPP) is rare, frequently relapsing and associated with limited evidence for targeted therapy. Vunakizumab is an anti-interleukin-17A monoclonal antibody, but data in paediatric GPP are scarce.

**Methods:**

We retrospectively reviewed three boys with recurrent GPP who received short-course vunakizumab during acute flares. Clinical characteristics, diagnostic details, treatment timelines, follow-up outcomes and Generalized Pustular Psoriasis Area and Severity Index (GPPASI) scores were collected.

**Results:**

A 13-year-old boy (87.5 kg) with ciclosporin-dependent recurrent GPP received vunakizumab 240 mg at weeks 0 and 2 with concomitant topical therapy; GPPASI decreased from 18 to 1, and no relapse was documented during approximately 6 months of follow-up. An 8-year-old boy (35 kg) received a single 120-mg administration; GPPASI decreased from 11 to 2, and no recurrent pustular eruption was reported during approximately 15 months of available follow-up, which was predominantly remote after the initial visit. Another 8-year-old boy (32 kg) with a febrile flare received two 120-mg administrations; GPPASI decreased from 14 to 2, and no relapse was documented during approximately 3 months of follow-up. No adverse events considered treatment-related were documented during the available observation periods.

**Conclusion:**

Short-course vunakizumab was temporally associated with rapid clinical improvement in three paediatric patients with acute GPP flares. Larger prospective studies with standardised follow-up are needed to evaluate efficacy, paediatric dosing, relapse prevention and long-term safety.

## 1 Introduction

Generalised pustular psoriasis (GPP) is a rare, severe and potentially life-threatening inflammatory dermatosis characterised by recurrent episodes of widespread sterile pustules on an erythematous background, often accompanied by systemic inflammation. Paediatric GPP is uncommon and frequently follows a relapsing course, while evidence to guide therapeutic selection remains limited (1, 2).

Current understanding of GPP pathogenesis highlights a central role for IL-36-driven innate immune activation. In addition, downstream IL-17-mediated inflammatory responses may amplify keratinocyte activation, neutrophil recruitment and pustule formation (3–6). This interaction provides a biological rationale for IL-17A-targeted treatment in selected patients with GPP.

Vunakizumab is a humanised monoclonal antibody targeting IL-17A and has demonstrated efficacy and acceptable tolerability in moderate-to-severe plaque psoriasis (7, 8). However, evidence supporting its use in GPP remains limited, particularly in children. Recently, an older patient with refractory GPP achieved rapid improvement after repeated induction doses of vunakizumab combined with acitretin (9). Unlike that combination-treatment setting, the clinical course following short-course vunakizumab during acute paediatric GPP flares has not been well described.

Here, we report three paediatric patients with recurrent GPP treated with short-course vunakizumab during acute flare episodes, with particular attention to rapid clinical response, GPPASI changes and short-term disease control.

## 2 Methods

### 2.1 Study design and patients

This was a retrospective descriptive case series involving three paediatric patients with recurrent GPP treated with vunakizumab during acute flare episodes at the Department of Dermatology, Wuhan Hospital of Integrated Traditional Chinese and Western Medicine (Wuhan No.1 Hospital).

Patients were included if they met the following criteria: (1) clinical diagnosis of GPP, supported by histopathological findings when available; (2) age younger than 18 years; (3) treatment with vunakizumab during an acute flare; and (4) availability of clinical records and follow-up information sufficient to evaluate short-term treatment response. Vunakizumab regimens were individualised in routine clinical care; no prespecified study protocol dictated paediatric dosing.

### 2.2 Data collection

Data on patient demographics (age, sex), clinical characteristics (body weight, height, disease duration, clinical manifestations, flare triggers), diagnostic evaluations (including laboratory, histopathological, and microbiological findings), and treatment details (previous therapies, vunakizumab regimen, concomitant treatment) were retrospectively collected from medical records.

### 2.3 Diagnostic assessment and exclusion of differential diagnoses

The diagnosis of GPP was established by experienced dermatologists based on recurrent generalised sterile pustules on an erythematous background, disease course, available histopathological findings, laboratory and microbiological results, and treatment response. Differential diagnoses, including acute generalised exanthematous pustulosis, pustular drug eruption, infection-related pustulosis, impetigo, fungal infection and subcorneal pustular dermatosis, were considered according to the clinical context and excluded where possible on the basis of the recurrent disease course, absence of a consistent new-drug trigger, microbiological findings, histopathology and clinical assessment. Genetic testing for *IL36RN* and other GPP-related genes was not available and was therefore considered a study limitation.

### 2.4 Outcome assessment

The primary descriptive outcome was change in disease severity following vunakizumab treatment, evaluated using the Generalized Pustular Psoriasis Area and Severity Index (GPPASI), a clinical disease measure used in GPP assessment (10). GPPASI scores at baseline and at the latest available photograph-supported clinical assessment were recorded for each patient. Scores were retrospectively assessed from available clinical photographs and medical records by the treating dermatologist; no independent blinded reassessment was performed.

### 2.5 Safety assessment

Available baseline and follow-up laboratory findings and clinically reported adverse events were extracted from the medical records. Adverse-event surveillance included outpatient documentation and remote follow-up where applicable. Systematic post-treatment laboratory monitoring was not available for all patients.

### 2.6 Statistical analysis

Given the descriptive nature of this study, no inferential statistical analysis was performed. Clinical characteristics, treatment regimens and treatment responses are presented descriptively.

### 2.7 Ethics

The study involving human participants was reviewed and approved by the Medical Ethics Committee of Wuhan Hospital of Integrated Traditional Chinese and Western Medicine (Wuhan No.1 Hospital), Wuhan, China (approval no. Kelun [2025] No. 151). Written informed consent was obtained from the legal guardians of all three paediatric patients for the publication of potentially identifiable clinical information and images included in this article.

## 3 Results

### 3.1 Patient characteristics

Three male paediatric patients with recurrent GPP were included, aged 8–13 years. All patients had experienced recurrent disease and had previously received conventional treatment, including ciclosporin. One patient had documented ciclosporin dependency, one experienced a prolonged recurrent disease course, and one presented with an acute febrile flare with generalized pustules. Clinical characteristics, treatment regimens and outcomes are summarised in **Table 1**.

**Table 1:** Clinical characteristics and treatment outcomes of three paediatric GPP cases treated with short-course vunakizumab.

Variable	Case 1	Case 2	Case 3
Age/sex	13 years/male	8 years/male	8 years/male
Weight/height	87.5 kg/173 cm	35 kg/height unavailable	32 kg/132 cm
Disease duration	>1 year	7 years	~5 years
Current flare	URTI-preceded generalised erythema and sterile pustules involving trunk, extremities, axillae and groin	Erythematous patches with needle-tip sterile pustules and fine scaling, mainly on face and trunk	Febrile generalised erythematous plaques with pustules, scaling and crusting involving trunk, extremities, scalp, face, folds, palms and soles
Systemic symptoms	No fever or other significant systemic symptoms	No fever or other significant systemic symptoms	Fever; resolved within 3 days after treatment initiation
Key laboratory/microbiology	WBC 8.43 × 10^9^/L; neutrophils 57.8%; CRP 0.76 mg/L; PCT 0.32 ng/mL; no evidence supporting infection-related pustulosis	WBC 7.40 × 10^9^/L; neutrophils 37.9%; CRP 1.44 mg/L; PCT 0.29 ng/mL; PAS/acid-fast stains and fungal culture negative	WBC 6.94 × 10^9^/L; neutrophils 55.9%; CRP 1.80 mg/L; skin-exudate culture negative; HBV/HCV/HIV/syphilis screening negative
Histopathology/genetics	Subcorneal neutrophilic pustules with psoriasiform epidermal hyperplasia and papillary dermal vascular changes; *IL36RN* testing unavailable	Subcorneal and intraepidermal neutrophilic pustules with epidermal hyperplasia and superficial inflammatory infiltrate; *IL36RN* testing unavailable	Neutrophilic aggregates in stratum corneum with psoriasiform epidermal hyperplasia and papillary capillary dilatation; *IL36RN* testing unavailable
Previous systemic therapy/status at initiation	Ciclosporin-dependent course; ciclosporin discontinued before vunakizumab; single ixekizumab administration with transient response	Ciclosporin 3.75–5 mg/kg/day in March 2024 with inadequate response; not receiving ciclosporin at vunakizumab initiation	Ciclosporin in July 2021 with partial/transient response; discontinued before current flare
Vunakizumab regimen	240 mg subcutaneously on 1 and 15 Jan 2026	120 mg subcutaneously once on 28 Mar 2025	120 mg subcutaneously on 26 Mar and 13 Apr 2026
Concomitant treatment	Urea, fusidic acid and fluticasone propionate creams; no systemic maintenance/rescue therapy recorded	No concomitant topical, systemic maintenance or rescue therapy recorded	Urea and dimeticone creams; no systemic maintenance/rescue therapy recorded
Clinical rationale	High body weight, active disease, ciclosporin dependency and transient previous ixekizumab response; adult experience and institutional practice	Lower body weight and cautious off-label approach; no further dose after rapid early improvement	Body weight, active febrile flare, paediatric safety considerations and institutional experience
Photograph-supported GPPASI	18 to 1 at week 2	11 to 2 at day 16	14 to 2 at follow-up on 13 Apr 2026
Follow-up	1 Jan–30 Jun 2026 (~6 months); outpatient records and remote follow-up	28 Mar 2025–30 Jun 2026 (~15 months); outpatient visit on 13 Apr 2025, then telephone follow-up	26 Mar–30 Jun 2026 (~3 months); last assessment by telephone
Relapse status	No clinical relapse documented	No recurrent generalised pustular eruption reported; no extended follow-up photographs available	No recurrent pustular eruption reported
Safety	No adverse event considered treatment-related documented; systematic laboratory follow-up incomplete	CBC normal at day 16; no adverse event considered treatment-related reported; later follow-up remote	No adverse event considered treatment-related documented; systematic post-treatment laboratory monitoring unavailable

AGEP, acute generalised exanthematous pustulosis; CRP, C-reactive protein; GPP, generalised pustular psoriasis; GPPASI, Generalized Pustular Psoriasis Area and Severity Index; HBV, hepatitis B virus; HCV, hepatitis C virus; PAS, periodic acid–Schiff; PCT, procalcitonin; URTI, upper respiratory tract infection; WBC, white blood cell count.

### 3.2 Case 1

A 13-year-old boy (weight 87.5 kg, height 173 cm) presented with recurrent erythema and pustules involving the trunk and extremities for more than 1 year. His disease first developed in December 2024, with diffuse erythema and widespread sterile pustules persisting for more than 1 month. Ciclosporin was initiated on 18 December 2024 at 150 mg twice daily, achieving disease control. The dose was reduced to 100 mg twice daily on 2 January 2025 and then to 75 mg twice daily on 6 January 2025. Subsequent tapering to 50 mg twice daily resulted in relapse. Re-escalation to 75 mg twice daily on 10 March 2025 improved the lesions, but another relapse occurred after ciclosporin was discontinued, indicating ciclosporin-dependent disease.

Approximately four months before the current flare, around September 2025, the patient received a single administration of ixekizumab. The exact dose and the interval from initial improvement to relapse were not clearly documented in the available medical records. The pustular eruption improved transiently, but the response was not durable, and relapse occurred before the current flare in January 2026.

One week before the current flare, an upper respiratory tract infection preceded recurrent generalised erythema and multiple sterile pustules involving the trunk, extremities, bilateral axillae and inguinal regions (**Figure 1A**). No fever or other significant systemic symptoms were observed. Histopathological examination revealed hyperkeratosis, parakeratosis, subcorneal neutrophilic pustules, psoriasiform epidermal hyperplasia, spongiosis, papillary dermal oedema, and proliferation and dilatation of superficial blood vessels. In the context of recurrent generalised sterile pustules on erythematous skin, these findings were considered compatible with GPP. Laboratory findings included a white blood cell count of 8.43 × 10^9^/L, neutrophils 57.8%, eosinophils 1.5%, C-reactive protein 0.76 mg/L and procalcitonin 0.32 ng/mL. No clinical or laboratory evidence supporting infection-related pustulosis was identified. The diagnosis of GPP was based on the recurrent disease course, characteristic eruption, compatible histopathology, exclusion of infection-related pustulosis and previous treatment response.

**Figure 1:**
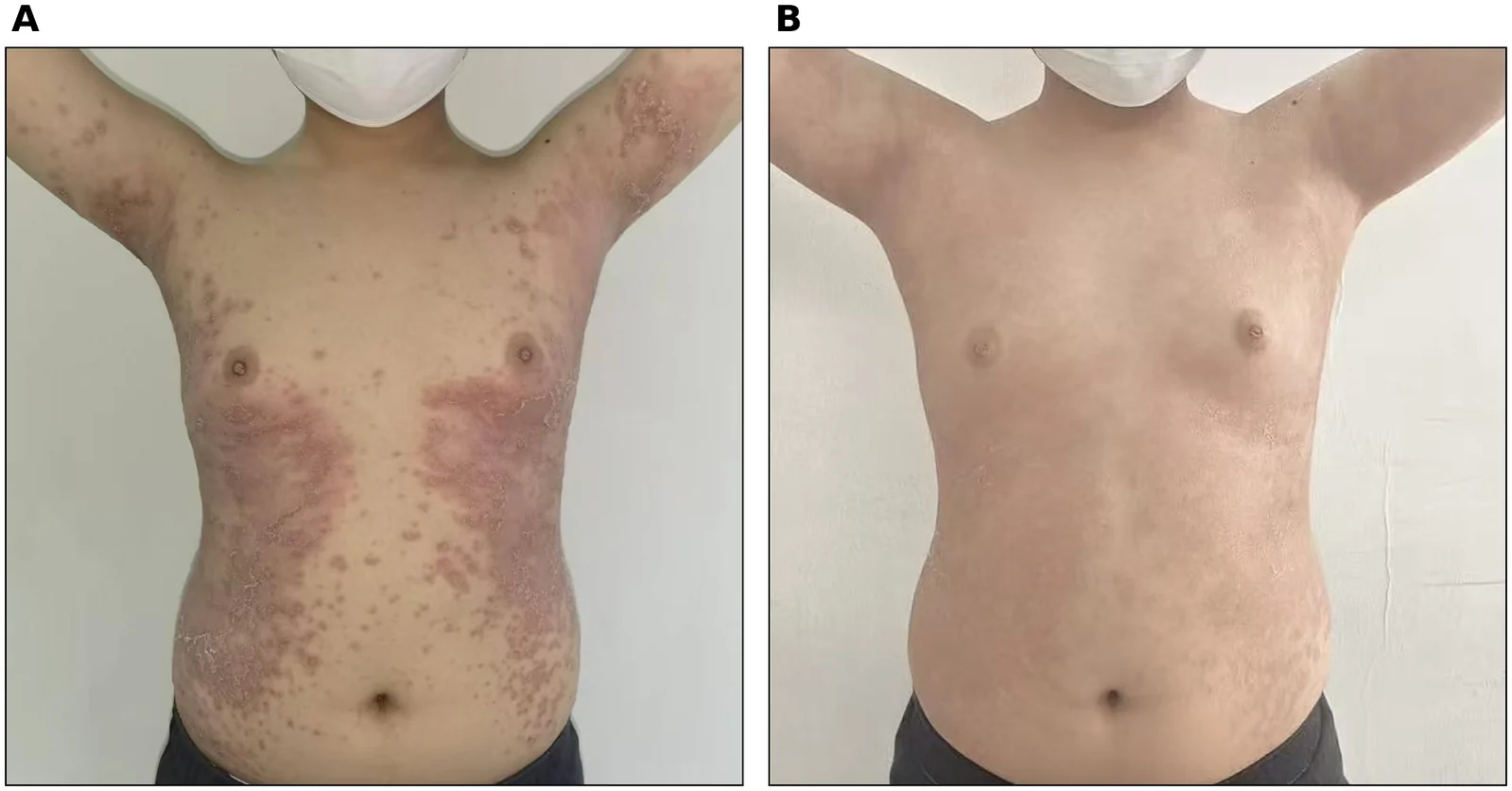
Clinical course of Case 1. **(A)** Generalised erythema and sterile pustules involving the trunk and flexural areas on 1 January 2026, before the first 240-mg subcutaneous dose of vunakizumab (GPPASI 18). **(B)** Near-complete pustule clearance with residual post-inflammatory hyperpigmentation on 15 January 2026, at week 2, when the second 240-mg dose was administered (GPPASI 1).

Ciclosporin had already been discontinued, and the response to the previous ixekizumab administration had been transient. Vunakizumab was therefore selected as another IL-17A-targeted option during the acute flare. Given the patient’s relatively high body weight (87.5 kg), active disease course, available adult clinical experience and institutional practice, a regimen of 240 mg subcutaneously at weeks 0 and 2 was used.

Vunakizumab 240 mg was administered subcutaneously on 1 and 15 January 2026, together with topical urea cream, fusidic acid cream and fluticasone propionate cream. Marked improvement was observed within 2 weeks, with near-complete pustule clearance and residual post-inflammatory hyperpigmentation (**Figure 1B**). GPPASI decreased from 18 on 1 January 2026 to 1 on 15 January 2026. Relapse status and adverse events were monitored through available outpatient records and remote follow-up until 30 June 2026. During approximately 6 months of available follow-up, no clinical relapse, injection-site reaction, infection, fever or other adverse event considered treatment-related was documented.

### 3.3 Case 2

An 8-year-old boy (weight 35 kg) with a 7-year history of recurrent GPP presented with an acute flare. He had received ciclosporin at 3.75 mg/kg/day on 18–19 March 2024, followed by dose escalation to 5 mg/kg/day from 20 to 28 March 2024 because of an inadequate response. Disease control remained unsatisfactory, and ciclosporin was not being administered when vunakizumab was initiated in March 2025.

At presentation, the patient had large erythematous patches predominantly involving the face and trunk, with densely distributed needle-tip-sized sterile pustules and fine scaling (**Figure 2A**). No fever or other significant systemic symptoms were observed. Histopathological examination demonstrated hyperkeratosis, epidermal hyperplasia, subcorneal and intraepidermal neutrophilic pustules, and superficial perivascular lymphocytic and neutrophilic infiltration. Periodic acid–Schiff and acid-fast staining were negative, and fungal culture with species identification yielded negative results. Laboratory findings before treatment included a white blood cell count of 7.40 × 10^9^/L, neutrophils 37.9%, eosinophils 0.15 × 10^9^/L, C-reactive protein 1.44 mg/L, procalcitonin 0.29 ng/mL, alanine aminotransferase 11 U/L, aspartate aminotransferase 20 U/L and serum creatinine 32 µmol/L. Chest computed tomography showed no significant abnormalities. The long recurrent course, characteristic morphology, compatible histopathology and negative microbiological investigations supported the diagnosis of recurrent GPP.

**Figure 2:**
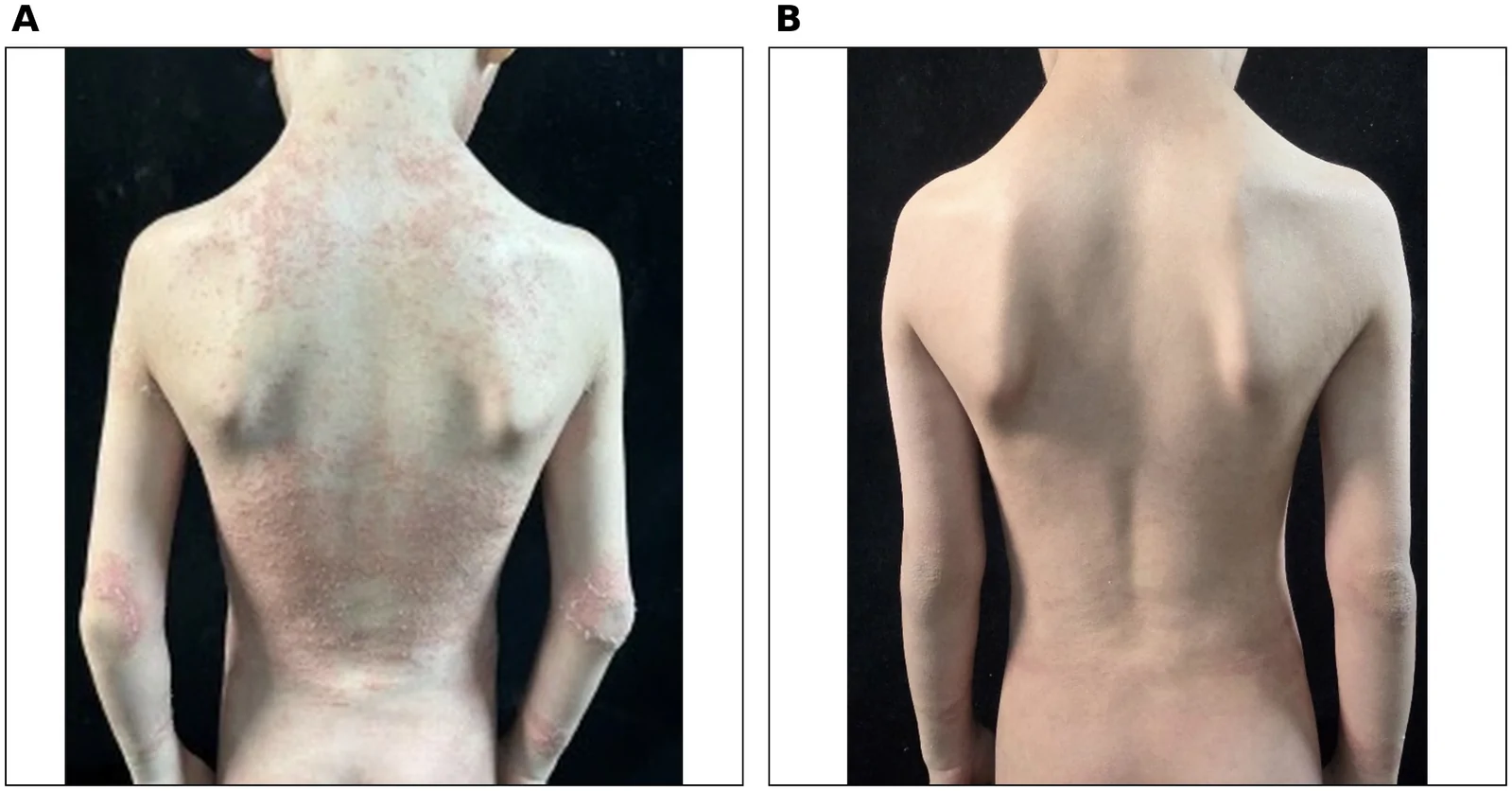
Clinical course of Case 2. **(A)** Erythematous patches with densely distributed needle-tip-sized sterile pustules and fine scaling on 28 March 2025, before the single 120-mg subcutaneous dose of vunakizumab (GPPASI 11). **(B)** Marked improvement of pustules with residual patchy erythema on 13 April 2025, 16 days after the single dose (GPPASI 2).

Considering the patient’s younger age, lower body weight and the need for a cautious approach to off-label biologic treatment, a single 120-mg dose of vunakizumab was selected.

The patient received a single subcutaneous injection of vunakizumab 120 mg on 28 March 2025. No concomitant topical therapy, systemic maintenance therapy or rescue treatment was recorded following vunakizumab. At the outpatient visit on 13 April 2025, the pustular eruption had markedly improved, with residual patchy erythema remaining on the trunk and extremities (**Figure 2B**); GPPASI decreased from 11 to 2. A complete blood count at this visit was within the normal range. Because of the rapid initial improvement and absence of early relapse, no additional dose was administered.

Follow-up extended from 28 March 2025 to 30 June 2026 (approximately 15 months), including the outpatient visit on 13 April 2025 and telephone interviews on 30 January, 30 March and 30 June 2026. During the remote contacts, the family reported no recurrent generalised pustular eruption and no injection-site reaction, infection, fever or other adverse event considered treatment-related. No clinical photographs were available from the extended remote follow-up, and no additional vunakizumab, systemic maintenance therapy or rescue treatment was recorded.

### 3.4 Case 3

Another 8-year-old boy (weight 32 kg, height 132 cm) had disease onset in 2021 and an approximately 5-year history of recurrent GPP. Previous treatment included ciclosporin 25 mg three times daily from 8 to 19 July 2021 and topical desonide cream, with only partial or transient responses. Ciclosporin had been discontinued before the current flare.

During the current flare, which began on 26 March 2026, the patient presented with fever and generalised erythematous plaques with scattered pustules of varying sizes, scaling and crusting. Lesions involved the trunk, extremities, scalp, face, intertriginous areas, palms and soles (**Figure 3A**). Histopathological examination demonstrated hyperkeratosis, parakeratosis, neutrophilic aggregates within the stratum corneum, psoriasiform epidermal hyperplasia, tortuous dilatation and congestion of papillary dermal capillaries, and superficial perivascular lymphocytic infiltration. Laboratory findings included a white blood cell count of 6.94 × 10^9^/L, neutrophils 55.9%, platelet count 276 × 10^9^/L, C-reactive protein 1.80 mg/L, alanine aminotransferase 9 U/L, aspartate aminotransferase 25 U/L, total bilirubin 15 μmol/L and serum creatinine 46.2 μmol/L. Pre-biologic screening was negative for hepatitis B virus, hepatitis C virus, HIV and syphilis, and chest computed tomography showed no significant abnormalities. Bacterial culture of skin exudate showed no growth of pathogenic organisms after 2 days of routine culture. The recurrent course, clinical morphology, compatible histopathology and negative microbiological investigation supported the diagnosis of recurrent GPP.

**Figure 3:**
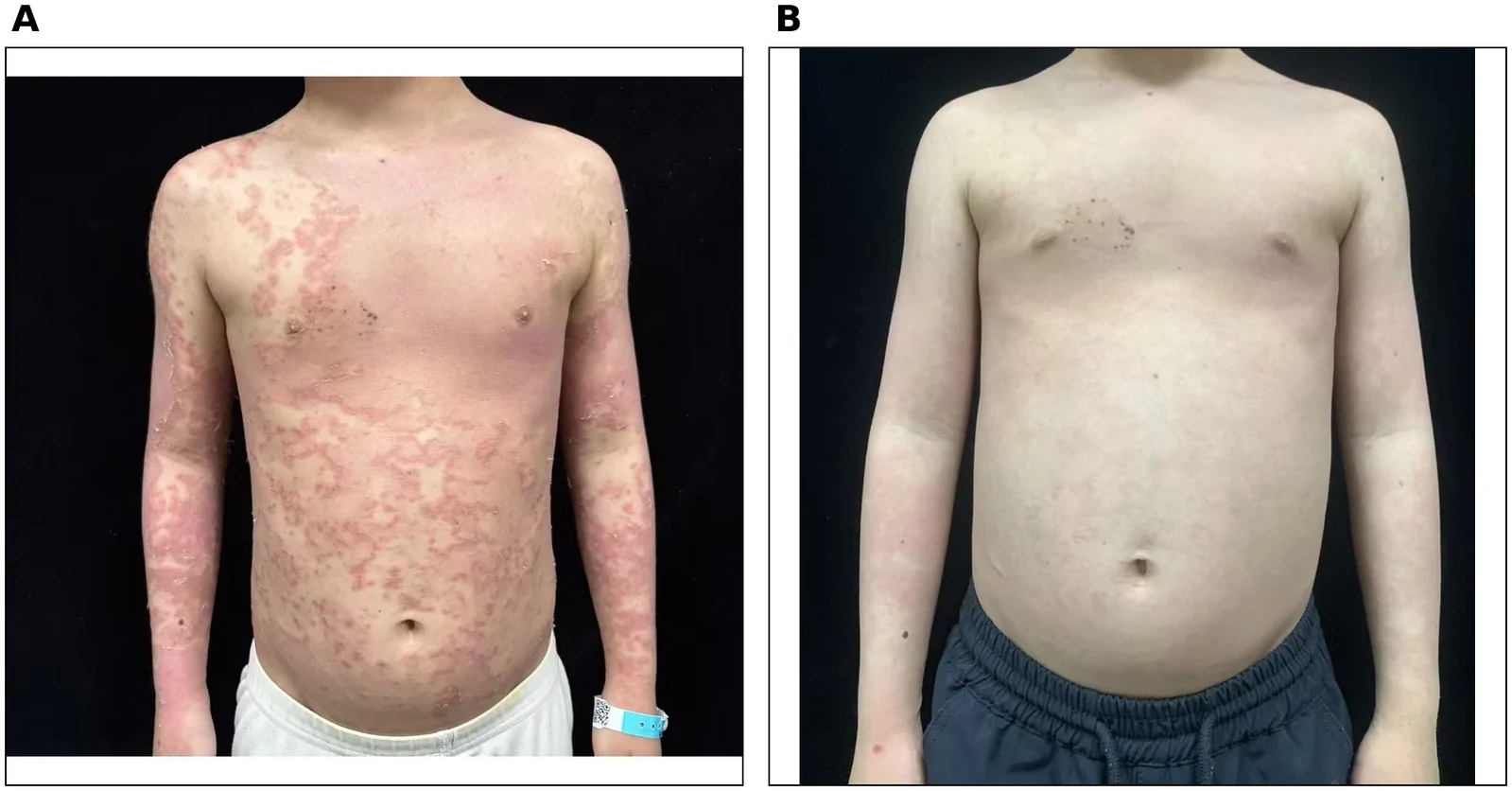
Clinical course of Case 3. **(A)** Febrile generalised pustular flare with erythematous plaques, scattered pustules, scaling and crusting on 26 March 2026, before the first 120-mg subcutaneous dose of vunakizumab (GPPASI 14). **(B)** Marked reduction in pustules and inflammatory erythema at follow-up on 13 April 2026, when the second 120-mg dose was administered (GPPASI 2).

Considering body weight, the active febrile flare, paediatric safety considerations and institutional clinical experience, a two-dose regimen of vunakizumab 120 mg was selected. Doses were administered on 26 March and 13 April 2026, together with topical urea cream and dimeticone cream. Fever resolved within 3 days. At the follow-up on 13 April 2026, the pustular eruption and erythema had markedly improved (**Figure 3B**), and GPPASI had decreased from 14 to 2. No concomitant systemic maintenance or rescue therapy was recorded after vunakizumab initiation.

The patient was followed until 30 June 2026, with the last assessment conducted by telephone interview. During approximately 3 months of available follow-up, no recurrent pustular eruption was reported, and no injection-site reaction, infection, fever or other adverse event considered treatment-related was documented. Systematic post-treatment laboratory monitoring was not available.

## 4 Discussion

In this retrospective case series of three paediatric patients with recurrent GPP, short-course vunakizumab administered during acute flares was temporally associated with rapid clinical improvement. GPPASI scores decreased from 18, 11 and 14 at baseline to 1, 2 and 2, respectively. No relapse was documented during the available follow-up periods, although follow-up methods differed and were partly remote. No adverse events considered treatment-related were documented, but safety interpretation is limited by incomplete systematic laboratory monitoring.

The rapid improvement observed in this series is consistent with previous reports of IL-17A blockade in pustular psoriasis and GPP across different age groups. Secukinumab has been reported to induce marked improvement in children with GPP (11), while ixekizumab has been reported to control severe pustular psoriasis in an adult (12). An older patient with refractory GPP also improved after vunakizumab combined with acitretin (9). The present cases differ in that they involved paediatric patients treated during acute flares with short-course vunakizumab and without concomitant systemic retinoid therapy.

Case 2 had the most distinctive clinical course: a single 120-mg administration was followed by marked initial improvement and no recurrent pustular eruption reported during approximately 15 months of available follow-up. One possible explanation is that IL-17A blockade during an acute flare may attenuate downstream neutrophilic inflammation and the reciprocal IL-17A/IL-36 inflammatory amplification described in experimental studies (3–6). This observation nevertheless remains hypothesis-generating. Extended follow-up was based on telephone interviews without contemporaneous photographs, and the case should not be interpreted as evidence that one dose is sufficient for paediatric GPP or that relapse prevention has been established.

Case 1 illustrates the challenge of ciclosporin-dependent recurrent paediatric GPP. Ciclosporin initially controlled the disease, but relapse occurred during dose reduction and after discontinuation. The response to a single administration of ixekizumab was transient, with incomplete documentation of its dose and response duration. Vunakizumab was therefore used as another IL-17A-targeted option during the subsequent acute flare in an attempt to regain disease control.

The three regimens were individualised on the basis of body weight, disease activity, prior treatment response, cautious off-label use and institutional experience. They were not derived from a validated paediatric GPP dosing protocol. Consequently, the observed responses cannot establish an optimal dose or schedule.

This study has several important limitations. First, only three patients were included, limiting generalisability. Second, the study was retrospective and descriptive; treatment regimens, concomitant topical therapies, follow-up methods and safety monitoring were not standardised. Third, GPPASI was reconstructed from available photographs and records by the treating dermatologist without independent blinded reassessment. Fourth, extended follow-up was partly remote, and no follow-up photographs were available for the prolonged observation period in Case 2. Fifth, the natural fluctuation of GPP and concomitant topical therapies may have contributed to improvement, so causality cannot be established. Genetic testing for *IL36RN* and other GPP-related genes was unavailable, and systematic post-treatment laboratory monitoring was incomplete. Finally, without a comparator group, no conclusion can be drawn regarding relative efficacy, optimal dosing, relapse prevention or long-term safety.

In conclusion, short-course vunakizumab was temporally associated with rapid clinical improvement in three paediatric patients with acute GPP flares. These observations are hypothesis-generating and should be interpreted cautiously because of the small sample size, retrospective design, heterogeneous treatment regimens and incomplete long-term safety monitoring. Larger prospective studies with standardised follow-up are needed to evaluate efficacy, paediatric dosing, relapse prevention and long-term safety.

## Data Availability

The data supporting the findings of this case series are not publicly available because they contain potentially identifiable information from paediatric patients. De-identified information may be considered upon reasonable request to the corresponding author, subject to ethical and privacy restrictions.
